# Supplementary material for: “When will it be over?” U.S. children’s questions and parents’ responses about the COVID-19 pandemic
Source: PLoS One. 2021 Aug 26;16(8):e0256692. doi: 10.1371/journal.pone.0256692 (PMC8389399; doi:10.1371/journal.pone.0256692)
Supplement: S1 File — (DOCX) [file pone.0256692.s001.docx]

**Stress and Coping**

Parents reported medium levels of anxiety and worry for themselves (*M_anxiety_* = 5.77, *SD_anxiety_* = 2.86, *M_worry_* = 6.25, *SD_worry_* = 2.60) and their families (*M_anxiety_* = 5.30, *SD_anxiety_* = 2.48, *M_worry_* = 5.76, *SD_worry_* = 2.33). However, parents reported that their child was less worried (*M* = 3.77, *SD* = 2.75) and less anxious (*M* = 3.41, *SD* = 2.78) than themselves or the family. Additionally, even with these levels of anxiety and worry parents reported that they (*M* = 7.00, *SD* = 1.95), their child (*M* = 7.36, *SD* = 2.00) and their family (*M* = 7.01, *SD* = 1.78) were coping relatively well. We fit a linear mixed-effects models predicting ratings of worry, anxiety and coping. For each model, we included the question type (parent, child or family, with child as the reference category) as a predictor. We also included a by-subject random intercept and a by-subject random effect for each contrast code (and allowed the random effects to correlate). We found an effect of question type, *F_worry_*(2, 346) = 138.96, *p* < .001, *F_anxiety_*(2, 344.52) = 109.15, *p* < .001, *F_coping_*(2, 346) = 10.28, *p* < .001. The ratings of worry and anxiety for their child were significantly lower than those for the parent, *t_worry_*(347) = 16.48, *p* < .001, *t_anxiety_*(346.09) = 14.14, *p* < .001, or the family, *t_worry_*(347) = 15.01, *p* < .001, *t_anxiety_*(345.23) = 13.96, *p* < .001. The ratings of coping for their child were significantly higher than those for the parent, *t*(347) = 3.69, *p* < .001, or the family, *t*(347.01) = 4.53, *p* < .001.

We attempted to collapse these measures into factors by conducting an exploratory factor analysis. Visual examination of the correlation matrix that 2 factors might be sufficient. See Figure A for a visualization of the correlation matrix. See Figure B to examine the scree plot. The scree-plot suggests that three factors have eigen values higher than 1. However, the three-factor solution included cross-loadings (i.e., items loading onto multiple factors) and thus it is not preferred. The two-factor solution did not include cross-loadings and so it is preferred. The two-factor solution included one factor that includes all the coping measures (which we will refer to as the coping factor), and one factor that includes all the worry and anxiety measures (which we will refer to as the stress factor). These two factors were negatively correlated (*r* = -0.37). Factor loading can be found in Table A. All items loaded onto at least one factor and no item loaded onto more than one factor. The coping factor explained 24% of the variance in the items, and the stress factor explained 39% of the variance (together explaining 63% of the variance in the items). We then fitted a confirmatory factor analysis based on the exploratory factor analysis to examine how well this model fit the data. We used a robust maximum likelihood estimator and a full information maximum likelihood to handle missing data. Our results show that this factor model does not fit the data, x2(26) = 495.65, p < .001, however, it does perform better than a baseline model, x2(36) = 1564.26, p < .001. Fit indices suggest that the model provides a poor fit for the data, CFI = .71, RMSEA = .28, SRMR = .10. The AIC for this model is 12549.29 and the BIC is 12657.152.

**Figure A.** Correlation among measures of anxiety, worry and coping for the parent, child, and family.


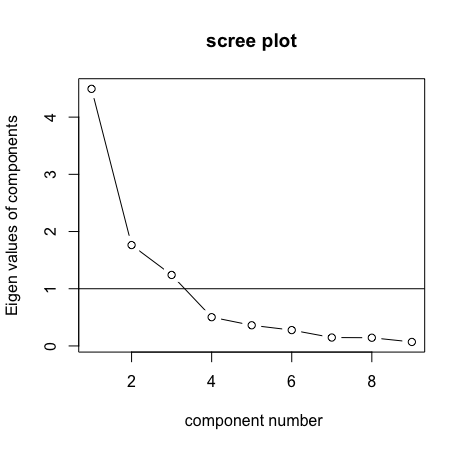


**Figure B.** Scree plot for the ratings of stress and coping.

**Table A.** Factor loadings for the worry, anxiety and coping measures. Numbers in Bold show factor loadings higher than .30, showing that the item loaded on to the factor.

|  | Coping factor | Stress Factor |  |
| --- | --- | --- | --- |
| Parent coping | | **0.80** | -0.03 |
| Child coping | | **0.64** | -0.15 |
| Family coping | | **1.00** | 0.04 |
| Parent worry | | 0.02 | **0.79** |
| Child worry | | 0.04 | **0.63** |
| Family worry | | 0.08 | **0.91** |
| Parent anxiety | | -0.13 | **0.72** |
| Child anxiety | | -0.07 | **0.61** |
| Family anxiety | | -0.04 | **0.82** |

In order to examine what variables predict stress and coping, we fitted a structural equation model. We included parents’ subjective SES, parent age, child’s age, parent biological knowledge, child biological knowledge, ratings of whether the parent had enough knowledge to answer the child’s questions, number of children in the household, number of adults in the household, and infection rate in their state of residence one week prior to their completion of the survey (April 7^th^, 2020; obtained from the CDC website: https://www.cdc.gov/mmwr/volumes/69/wr/mm6915e4.htm?s_cid=mm6915e4_x) as predictors of both stress and coping. We used a robust maximum likelihood estimator and a full information maximum likelihood to handle missing data. This model was not a good fit for the data, χ^2^(89, N = 348) = 774.19, *p* < .001, CFI = 0.671, TLI = 0.567, RMSEA = 0.149 [0.140, 0.158]. The only predictor of family stress was the number of adults in the household, with more adults increasing stress, *b* = 0.395, *z* = 2.08, *p* = .038. As parent’s subjective SES increased coping scores increased, *b* = 0.17, *z* = 2.92, *p* = .003. Finally, as parent’s ratings of having enough knowledge increased, coping scores increased, *b* = 0.215, *z* = 2.07, *p* = .038.

**Coping strategies.** We analyzed the coping strategies that parents reported. The coding scheme for these responses can be seen in Table B. The most common coping strategy was to engage in different activities such as walking, exercising, playing games, cooking, or watching television or movies (*n* = 283, 81.8%). Many parents also reported spending time interacting with friends and family (*n* = 184, 53.2%) either in their home or virtually. Parents also reported trying to keep a consistent schedule or regular routine (*n* = 77, 22.2%). Some also reported engaging in COVID-related coping strategies (*n* = 53, 15.3%) such as washing their hands, social distancing, watching (or avoiding) news related to the pandemic, or having health-related conversations with their family members. Some parents reported engaging in activities related to education (*n* = 47, 13.6%) such as homeschooling their children or learning new skills. Some parents used prayer or other religious activities to cope (*n* = 25, 7.2%). A small number of parents reported having no coping strategies (*n* = 13, 3.7%), treating the pandemic as a vacation (*n* = 5, 1.4%), or acting like nothing was happening (*n* = 2, 0.5%). Nine parents (2.6%) reported other coping strategies not captured in our coding scheme.

The responses to whether there was anything else parents wanted to share with us were very similar to the responses about coping strategies. The coding scheme for these responses is shown in Table C. About half of the parents said they did not have any other information (*n* = 165, 47.4%). Again, many parents reported spending time in activities (*n* = 16, 4.6%), interactions with family and friends (*n* = 18, 5.2%), and COVID-related activities (*n* = 38, 11.0%). Some parents also reported keeping a routine (*n* = 9, 2.6%), praying or engaging in other religious activities (*n* = 5, 1.4%), or engaging in educational activities (*n* = 4, 1.2%). Additionally, some parents reported feeling uncertain about the future (*n* = 5, 1.4%) and expressed financial concerns (typically related to job loss; *n* = 4, 1.2%). Finally, some parents used this opportunity to share how they were coping. Some said they were not coping well with the pandemic (*n* = 12, 3.5%), while other said that they were coping well (*n* = 14, 4.0%). Some parents mentioned they were “winging it” or coping as best they could (*n* = 20, 5.8%). Thirty parents (8.7%) gave other idiosyncratic answers.

**Table B. Coding of coping strategies.**

| Code | Description | Example | Kappa |
| --- | --- | --- | --- |
| No strategies | Parents report they have no coping strategies | “No”  “None” | 0.59 |
| Acting like nothing has happened | Reponses that describe acting as if the pandemic was not happening | “We are going through life as nothing has happened”  “We are acting like nothing is happening” | 0.66 |
| Treating it like a vacation | Responses that describe treating the pandemic as a vacation. | “We are treating like a vacation”  “it is pretty much like an early summer vacation” | 0.75 |
| Keeping a regular schedule or routine | Responses that describe some kind of routine or doing activities on a recurring basis | “We keep a daily routine”  “We have developed a daily routine”  “…daily…”  “…everyday…” | 0.80 |
| COVID Related | Responses that relate to the COVID-19 such as washing hands, talking about health, following news reports, social distancing. | “we are staying home”  “more conversations about health in general”  “we wash our hands a lot”  “actively avoiding over saturation of COVID coverage”  “reading news about corona”  “observing social distancing” | 0.81 |
| Activities | Responses about engaging in different activities such as  games, watching TV, exercising, walking, being outside, cooking, reading, etc. | “we are playing games”  “we are watching movies or series on Netflix”  “we make sure to get a lot of exercise”  “we go for walks”  “we are reading a lot”  “creative projects” | 0.92 |
| Social Interactions | Responses about different ways the families are interacting with people either inside or outside the home | “we are having much more family time”  “using social media to connect with other family and friends” | 0.83 |
| Education | Responses about educating the child or learning new skills. | “We are doing school work at home”  “we learn new things that are not taught in school” | 0.82 |
| Prayer/Church | Responses about religion or religious activities | “daily devotional together”  “we pray at home” | 0.98 |
| Other | Responses that did not fit into any of the above categories. | “Working from home”  “using the internet” | 0.53 |

**Table C. Coding of other information**

| Code | Subcategories | Description | Example | Kappa |
| --- | --- | --- | --- | --- |
| No other information |  | Parents report they have nothing else to tell us | “Nothing else comes to mind”  “Not at this time” | 1.00 |
| Keeping a regular schedule or routine |  | Responses that describe some kind of routine or doing activities on a recurring basis | “…we make sure to keep out trips to once ever week or two…”  “we have a little celebration every Saturday” | 0.87 |
| Prayer or church |  | Responses about religion or religious activities | “we pray daily for all”  “praying for others” | 0.89 |
| Uncertainty about the future |  | Responses describing how individuals are not sure of what is to come | “the biggest question, and the one we cannot answer, is ‘how long?’…”  “there is a lot of uncertainty right…” | 0.72 |
| Financial insecurity |  | Responses that relate to losing jobs, not having money, not collecting unemployment, or other financial uncertainty caused by the COVID-19 pandemic. | “financial times are scary…”  “just been tough losing income…”  “we are both furloughed, things are very rough for us financially…” | 1.00 |
| COVID related |  | Responses that relate to the COVID-19 such as washing hands, talking about health, following news reports, social distancing | “staying safe by wearing mask and gloves to be well cover…”  “…discuss what happens if anyone gets sick…” | 0.73 |
| Activities |  | Responses about engaging in different activities such as  games, watching TV, exercising, walking, being outside, cooking, reading, etc. | “we try to come up with more games”  “using our pool”  “go for scavenger hunts” | 0.87 |
| Social interaction |  | Responses about different ways the families are interacting with people either inside or outside the home | “we are trying to just enjoy each other’s company…”  “spending time with each other” | 0.88 |
| Education |  | Responses about educating the child or learning new skills | “homeschooling is hard”  “we also homeschool…” | 0.86 |
| Coping | Not coping well | Responses that indicate the parent, child or family is not coping well with the COVID-19 pandemic | “my mom isn’t handling it well”  “I feel like I’ve let me family down”  “going crazy staying inside” | 0.74 |
|  | Coping well | Responses that indicate the parent, child, or family is coping well given the COVID-19 pandemic | “I think my kids are coping well…”  “We are doing pretty good considering”  “I think we’re doing very well” |  |
|  | Coping the best we can | Responses that indicate the parent, child, or family is doing what they can to cope, taking it day by day, trying to stay positive, or winging it | “it is just a day by day thing”  “just doing the best we can…”  “Not the easiest thing to do most days, but we are managing” |  |
| Other |  | Responses that did not fit into any of the above categories. | “yes who has it in my neighborhood”  “sickness is part of life. Just accept that and keep moving” | 0.70 |
