# Supplementary material for: “When will it be over?” U.S. children’s questions and parents’ responses about the COVID-19 pandemic
Source: PLoS One. 2021 Aug 26;16(8):e0256692. doi: 10.1371/journal.pone.0256692 (PMC8389399; doi:10.1371/journal.pone.0256692)
Supplement: Attachment — Submitted filename: Response to reviewers 2.docx [file pone.0256692.s003.docx]

Reviewers' comments:

**Reviewer #1:**

**Thank you for adressing the points of my first feedback.**

We thank the reviewer for their positive feedback and their thorough read of our manuscript.

**I have still concerns regarding the following points:**

**Statistical analysis and criteria for model fit:
I suggested to include model fit in order to test, if the measurement models of coping and stress fit the data well. This is reported with goodness of fit values such as the χ2/df (ratio chi-square and degrees of freedom), CFI (comparative fit index), the RMSEA (root mean square error of approximation), and the SRMR (Standardised Root Mean Square Residual). CFI values of .90 or greater indicate adequate, CFI values of .95 and greater indicate good fit. RMSEA values of .08 or less and .05 or less indicate adequate and good fit, respectively (Hu & Bentler, 1999). I found these measurement models in your R code, but not the documention of the values in the paper.**

Given that this measurement model is not the main topic of our paper, we provide this information in the supplemental materials (S1 file). In this file, we previously only included fit indices for the structural equation model predicting the factor scores. We did not report fit indices on the factor analysis as this analysis was exploratory, and so we did not want to give the impression that we were testing how well a theoretically motivated model fit the data. To attempt to address the reviewer’s concern we have clearly labelled the explanatory factor analysis as such in the paper and the supplemental materials. Then, we detail how we fitted a confirmatory factor analysis in order to see how well the exploratory factor analysis fitted the data, and report these values. Because the measurement of stress and coping is not the main concern of our paper, we include all of this information in the supplemental materials. We apologize for misunderstanding which model the reviewer was referring to in their first review.

**You instead reported the Hosmer and Lemeshow goodness of fit test (table 6), that tests the goodness of fit of logistic regression models. You applied Generalized linear mixed-effects models (with a binomial link function). As to my knowledge GLMM need other goodness-of-fit methods (**[**https://www.frontiersin.org/articles/10.3389/fpsyg.2021.666182/full**](https://www.frontiersin.org/articles/10.3389/fpsyg.2021.666182/full)**). In my opinion this is crucial to assess the quality of your models where you try to «predict» some outcomes.**

We report the Hosmer and Lemeshow test as it provides a chi-square for how the model fits the data by comparing the number of observations the model expects to the observed observations. This is a test often used for logistic regression models (Hosmer, Lemeshow, & Sturdivant, 2013). The paper the reviewer references suggest that many articles report the AIC. We previously did not report the AIC as this statistic is not interpretable on its own. AIC is only meaningful in model selection, where it can be used as a criterion for selecting between different models. Given that we are not comparing different models in our paper, the AIC is not interpretable. In order attempt to address the reviewers’ concerns we now also report the AIC and BIC in Table 6, but do not attempt to interpret them.

Hosmer DW, Lemeshow S, Sturdivant RX. Applied Logistic Regression, 3rd Edition. 2013. New York, USA: John Wiley and Sons.

**Regarding the wording I have an additional concern: You have only cross-sectional data that doesn’t allow to «predict» an outcome.**
We have revised the wording throughout the manuscript and now use “associated with” instead of “predict” when relevant.

**On this background I assess the statistical analysis as not appropriate and rigorous enough.**

We hope some of the changes we made highlight the appropriateness and rigor of our analyses.

**Discussion
This section is still confusing as you do not answer your research questions in a structured way.**

We have attempted to provide more structure to the discussion section. We have reorganized the information and added subheadings to attempt to provide more structure. We also added more discussion of some of the results in order to relate the findings to each other (last comment by the reviewer). Please see revised discussion section.

**The distinction between reported and targeted questions is not mentioned**

We now mention that we had both reported and targeted questions, and the reason why we examined each. We also discuss the results of both types of questions and clearly label which we are discussing.

**and you report results, that are not mentioned before (p. 24, line 437 – 439).**

This information was included in the results section. In page 24 of the previous manuscript, we said “Additionally, shielding does not seem related to how the child is coping with the pandemic, but rather to parents’ beliefs about the appropriate age to expose children to this information.” In the results section (page 23), we mention how many parents reported shielding their children because “they thought they were not old enough to understand what is going on (n = 83, 23.8%)” (line 402-403). In that same page we also mention how our other predictors, including child coping did not predict shielding. We have attempted to clarify this information on the discussion section.

**This part needs a rigorous revision that stringently and logically puts the results in relation to each other.**

We have attempted to revise the discussion section as suggested by the reviewer. We hope the new discussion section addresses the reviewer’s concerns and shows the novelty of our findings.
